# Potential of aquatic oomycete as a novel feedstock for microbial oil grown on waste sugarcane bagasse

**DOI:** 10.1007/s11356-018-3183-8

**Published:** 2018-09-28

**Authors:** Alok Patel, Leonidas Matsakas, Parul A Pruthi, Vikas Pruthi

**Affiliations:** 1 Biochemical Process Engineering, Division of Chemical Engineering, Department of Civil, Environmental and Natural Resources Engineering, Luleå University of Technology, SE-97187 Luleå, Sweden; 2 Molecular Microbiology Laboratory, Biotechnology Department, Indian Institute of Technology Roorkee (IIT-R), Roorkee, Uttarakhand 247667 India

**Keywords:** Oleaginous mold, Biodiesel, Semi-solid-state fermentation, Fluorescence microscopy, Lipid extraction, Fatty acid methyl esters

## Abstract

Biodiesel production from vegetable oils is not sustainable and economical due to the food crisis worldwide. The development of a cost-effective non-edible feedstock is essential. In this study, we proposed to use aquatic oomycetes for microbial oils, which are cellulolytic fungus-like filamentous eukaryotic microorganisms, commonly known as water molds. They differ from true fungi as cellulose is present in their cell wall and chitin is absent. They show parasitic as well as saprophytic nature and have great potential to utilize decaying animal and plant debris in freshwater habitats. To study the triacylglycerol (TAG) accumulation in the aquatic oomycetes, the isolated water mold *Achlya diffusa* was cultivated under semi-solid-state conditions on waste sugarcane bagasse, which was compared with the cultivation in Czapek (DOX) medium. *A. diffusa* grown on waste sugarcane bagasse showed large lipid droplets in its cellular compartment and synthesized 124.03 ± 1.93 mg/gds cell dry weight with 50.26 ± 1.76% *w*/*w* lipid content. The cell dry weight and lipid content of this water mold decreased to 89.54 ± 1.21 mg/gds and 38.82% *w*/*w*, respectively, when cultivated on standard medium Czapek-Dox agar (CDA). For the fatty acid profile of *A. diffusa* grown in sugarcane bagasse and CDA, in situ transesterification (IST) and indirect transesterification (IDT) approaches were evaluated. The lipid profile of this mold revealed the presence of C_12:0_, C_14:0_, C_16:0_, C_18:0_, C_18:1_, C_18:2_, C_20:0_, and C_21:0_ fatty acids, which is similar to vegetable oils. The biodiesel properties of the lipids obtained from *A. diffusa* satisfied the limits as determined by international standards ASTM-D6751 and EN-14214 demonstrating its suitability as a fuel for diesel engines.

## Introduction

Biodiesel, as one of the best alternative fuels for transportation and industrial purposes, has various advantages over petrodiesel, such as origination from renewable and indigenous feedstock, low toxicity, better lubricity, superior flash point, and biodegradability (Patel et al. 2017b). Biodiesel consists of a mixture of fatty acid methyl/ethyl esters derived from TAG. In recent years, much attention has been paid to biomass-based biofuels as the availability of petroleum-based resources continues to decline with continuously increasing energy demands (Patel et al. 2016). Among the various biomass-based biofuels, biodiesel is considered an important renewable source to the rapidly depleting fossil fuels, contributing to the development and adoption of effective alternatives. Vegetable oils are still the leading source for biodiesel production, but they possess limitations regarding their attainability at a competitive price, which decreases the interest of biodiesel as a competitive alternative to petroleum-based fuel (Singh and Singh 2010). Therefore, other oil sources have been increasingly explored in order to meet the increasing demand for biodiesel. In this regard, important considerations have been made for the sustainable production of fatty acids from microbial sources. Microbial sources for lipid production have many advantages over their counterparts, including higher lipid yield, devoid of any seasonal and climatic change, less labor intensive, and easy scale up (Huang et al. 2013). Lipids produced by microorganisms, involving yeasts, bacteria, fungi, and algae, so-called single cell oil (SCO), are indicated as a promising feedstock for biodiesel production because of their similar fatty acid profile to vegetable oils (Ratledge 2004). These microorganisms can utilize organic carbon sources irrespective of their origin to accumulate oils in their cellular compartment, so-called lipid droplets (LDs), and it has already been reported that the productivity of many microorganisms is higher than that of oil-producing crops (Knothe and Razon 2017). In this study, a water mold was explored as a novel feedstock for potential microbial oil production.

The oomycetes are endemic to fresh water and are a group of diploid eukaryotes commonly known as water molds; moreover, they can thrive anywhere in nature with the least supply of food than any other microorganisms (Diéguez-Uribeondo et al. 2004). The most common oomycetes belong to the family Saprolegniaceae, which includes a wide variety of water molds, showing saprophytic nature as they grow on animals and plant debris or as parasites on plants and animals (Diéguez-Uribeondo et al. 2009). Saprolegniaceae also includes many pathogenic species, such as *Phytophthora infestans,* the causal agent of potato late blight (Diéguez-Uribeondo et al. 2009). Oomycetes show many morphological characteristics similar to fungi; therefore, they are classified within the kingdom of Fungi; however, phylogenetic studies revealed similarities with diatoms and chromophyte algae (Johnson et al. 2000). The absence of chitin and presence of cellulose in the cell wall of oomycetes are the distinguishing features from true fungi, but they share some common infection strategies with fungi (Badreddine et al. 2008). The lipid extraction is a crucial step in biodiesel production, and it is totally dependent on the cellular structure of the oleaginous microorganism from which the lipids are extracted (Leung et al. 2010). However, various methods have been applied for algal cell disruption and efficient lipid extraction (McMillan et al. 2013). The cell wall of oomycetes has shown similarity with the algal cell wall due to the presence of cellulose (Johnson 1956).

**Fig. 1:**
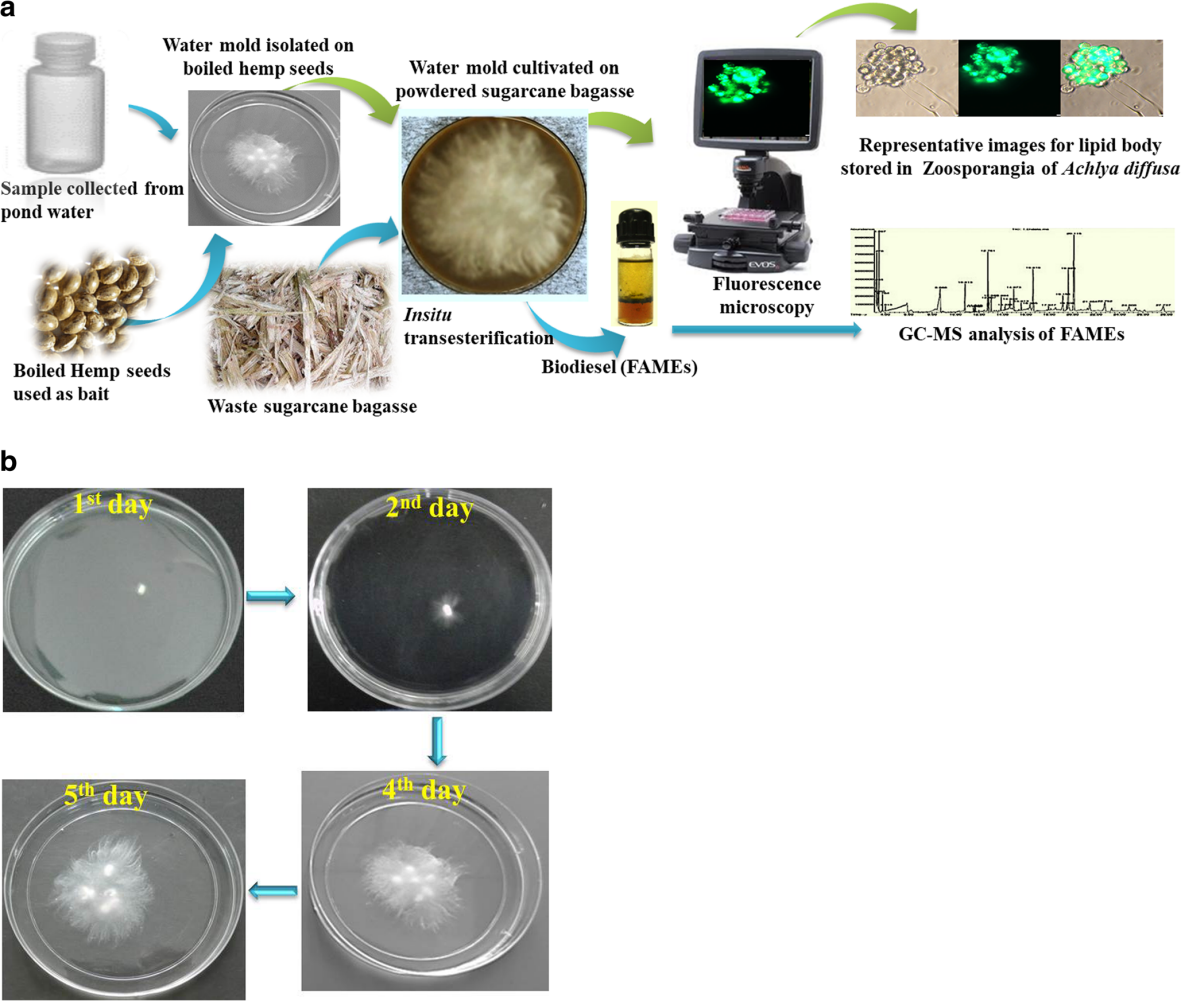
Schematic diagram of the isolation of water mold and biodiesel production (**a**). Growth at different time intervals of *A. diffusa* on boiled hemp seed during isolation process (**b**)

In the present study, waste sugarcane bagasse was used as solid medium for the growth of the isolated water mold *A. diffusa*. Various non-edible lignocellulosic biomasses have been utilized for the third-generation biofuel production since the last decade (Patel et al. 2016). Among those, sugarcane is the most promising biomass for renewable energy as it consists of two different types of biomass, bagasse, and cane trash (Loh et al. 2013). Bagasse is the leftover after milling of sugarcane, which contains lignocellulosic biomass, including a small amount of sugars, wax, and minerals (Loh et al. 2013). Solid-state fermentation is a common method to cultivate filamentous fungi, in which the sugars are provided without prior extraction (Pensupa et al. 2013). In order to maximize the utilization of sugars from solid substrates, little amount of water was added to the substrate, which is now called semi-solid fermentation (Pandey et al. 2000; Dey et al. 2011). The present investigation ensures the high growth rate and maximum lipid production by isolated water mold (Fig. 1).

The high cost associated with biodiesel production from oleaginous microorganisms is the major obstacle to use microbial sources as feedstocks as it involves cultivation, harvesting, lipid extraction, and transesterification (Patel et al. 2016). Disruption of cells for lipid recovery from biomass is an essential process before the transesterification reaction and the required drying/dewatering step before extraction is a comparatively energy-intensive process and a major bottleneck for cost-effectiveness of biodiesel production (McMillan et al. 2013). Traditional methods (BD and Folch method) for lipid extraction involve chloroform and methanol which are effective only at a lab-scale level (Halim et al. 2012). In this study, two different procedures for transesterification were evaluated to determine the fatty acid methyl ester (FAME) profile. The first one was the in situ transesterification, with direct conversion of fatty acids without lipid extraction. In the second procedure, the lipids were extracted from dry biomass followed by the transesterification of lipid into FAME. The direct or in situ transesterification process eliminates cell drying and lipid extraction steps, which gives a higher yield of fatty acid methyl esters than that obtained after conventional methods (Cheirsilp and Louhasakul 2013).

## Methods

### Materials

All solvents such as methanol, *n*-hexane, chloroform, diethyl ether, and acetic acid were of analytical grade and procured from Sigma-Aldrich (St. Louis, MO, USA). NaCl, KCl, anhydrous sodium sulfate, Czapek-Dox medium, and H_2_SO_4_ were obtained from Merk, Germany. TAG standard (Triolein) for thin layer chromatography (TLC) was purchased from Sigma-Aldrich (St. Louis, MO, USA). BODIPY_505/515nm_ for fluorescent staining purposes was procured from Invitrogen (Life Technology, USA). Sugarcane bagasse, used for semi-solid-state fermentation in this study, were obtained from local market.

### Isolation of water mold

The water sample was carried aseptically from the pond area of Roorkee (29°52′29.49″N 77°53′23.74″E) to the laboratory, avoiding any leakage. The bottles were slightly shaken and the aliquots of 15-ml samples were poured into sterilized Petri plates. Boiled hemp seed cotyledons (4–5) floated over it at room temperature. After 48–72 h, hyphal growth appeared on the baits, which was thoroughly washed with a thin stream of sterilized water. Pure cultures were obtained by picking up a small tuft of mycelium and grown on Czapek-Dox agar media at 25 °C. Isolated water mold was maintained on Czapek-Dox agar (CDA) slants at 4 °C. To prevent bacterial growth, streptomycin (50 μg/ml) was added to the media.

### Identification and characterization of the isolated water mold

The pure culture was examined for colonial growth and morphological features. The sporangial structures were observed in an inverted light microscope, and the genera was identified on the basis of sporangial discharge. The specific characteristics of the isolated water mold, such as the formation of sporangia; development, orientation, specific shape, and size of oögonia/antheridia; the structure of oöspores; and formation and structure of gametangia, were studied to identify the species.

### Cultivation of the isolated water mold for biomass and lipid production using semi-solid-state fermentation of sugarcane bagasse

Sugarcane bagasse (50 g) was desiccated in a hot air oven at 60 °C for 24 h and then crushed using a kitchen grinder to 1–2 mm particles in size. Ground sugarcane bagasse was stored at room temperature. Petri dishes were used to monitor the aerobic growth of *A. diffusa* aseptically, and an appropriate amount of substrate was carefully selected in order to gain an ideal aeration of the growth media, achieving a stable growth of the water mold. Crusted sugarcane bagasse (10 g) was mixed with 20 ml of mineral salts (MS) solution and placed in Petri dishes. The MS solution was composed of (in g/l): NH_2_SO_4_, 2.0; KH_2_PO_4_, 1.0; MgSO_4_·7H_2_O, 0.5; FeSO_4_·7H_2_O, 0.01; ZnSO_4_·7H_2_O, 0.01; MnSO_4_·4H_2_O, 0.001; CuSO_4_·5H_2_O, 0.0005; CaCl_2_·2H_2_O, 0.2. The pH of the MS solution was adjusted to 6.5. Hyphal fragments from stored cultures were aseptically transferred to Petri plates and incubated at 25 °C for 8 days. The cultivation of water mold on Czapek-Dox agar was used as a control experiment. The estimation of cell dry weight in solid-state fermentation is difficult due to penetration of fungal hyphae into substrate and strong binding to the solid substrate particles. However, in the case of semi-solid-state fermentation, the complete recovery of fungal biomass from the substrate is usually easy. Hence, mycelia grown in the Petri dishes were firstly harvested and washed several times with distilled water to remove solid particles attached to mycelia. Mycelia were then dried between paper towels to remove water, followed by freezing in liquid nitrogen, lyophilization, and finally weighing to estimate dry mass. The total residual carbohydrate amount in the samples was estimated by the standard phenol-sulfuric acid method. Time course experiments were performed in several Petri plates and the entire sample from each plate was utilized for estimatation of cell dry weight, lipid content, and amount of residual carbon sources.

### BODIPY staining and fluorescence microscopy

The stock solution of BODIPY_505/515nm_ (1 mg/ml) was prepared in DMSO. Cultures cultivated on sugarcane bagasse were washed twice with distilled water, and fresh distilled water was added to Petri plates. The 10 μl BODIPY stock solution (10 μg/ml) was then added and the solution was mixed thoroughly. After 5 min of incubation in the dark, the cell suspension was washed four times with 0.9% saline distilled water. The imaging was performed on a digital fluorescence microscope (EVOS FL Cell Imaging System, Thermo Fisher Scientific, USA) using the LED-based green fluorescence cube. The lipid droplets within the cellular compartments of isolated water mold were examined by ImageJ 1.48a software.

### Lipid extraction and gravimetric determination

The total cellular lipid from water mold was attained by extraction with chloroform/methanol (2:1, *v*/*v*) with certain modifications (Bligh and Dyler 1959). Dried samples were vigorously mixed with distilled water for 15 s. The cell suspension was subjected to sonication at 40 Hz for 5 min and after that, 10 ml of chloroform/methanol (2:1; *v*/*v*) was added to the suspension. A sintered glass funnel was used to filter the extract. Five milliliters of 0.034% MgCl_2_ was poured into the extract, which was then centrifuged at 3000 rpm for 5 min. The upper aqueous layer was removed, and the lower organic phase was washed with 1 ml of 2 N KCl/methanol (4:1; *v*/*v*). After washing, the suspension was centrifuged again at 3000 rpm for 5 min and washed repeatedly with 5 ml of chloroform/methanol/water; 5:48:47; per vol. until the phase boundary became clear. The bottom-most chloroform layer was shifted to a new pre-weighed screw cap vial. The total lipid yield, expressed as milligram lipid per gram dry substrate (mg/gds), was determined gravimetrically (Cheirsilp and Kitcha 2015).

### Transesterification of extracted lipid

The lipids were transesterified into fatty acid methyl esters (FAMEs) by two different methods viz. in situ transesterification or direct transesterification (without preliminary extraction steps) and indirect transesterification (two-step transesterification), in which lipid was extracted from biomass followed by transesterification.

#### In situ transesterification

The in situ transesterification reaction was processed by using dried biomass of water mold and 6% methanolic H_2_SO_4_. Briefly, 100 mg of dried biomass was mixed with methanolic sulfuric acid (6%) in a GC-MS vial (Teflon coated) and vortexed well. The vial was then kept in a water bath to proceed the transesterification reaction at 90 °C for 1 h. After completion of the reaction, the vial was cooled down to room temperature and mixed with hexane/water (2:1) to extract the methyl esters. After centrifugation at 3500×*g*, the upper hexane layer, containing esters, was transferred to a new pre-weighed vial and washed with distilled water several times followed by dehydration with anhydrous sodium sulfate.

#### Indirect transesterification (two-step transesterification, indirect transesterification)

The total cellular lipids were firstly extracted from dry biomass of water mold using a modified method of Bligh and Dyler (1959) as described in the “Lipid extraction and gravimetric determination” section. The extracted lipids were then transesterified using the same protocol as mentioned in the “In situ transesterification” section.

### Thin layer chromatography and Fourier-transform infrared of extracted lipid from *A. diffusa*

The total lipid analysis for TAG was carried out by thin layer chromatography (TLC) and Fourier-transform infrared (FTIR) spectroscopy. The chromatograms for TLC were developed by using G-60 F254 plates, Merck, India (0.25 mm thick silica gel) with hexane/diethyl ether/acetic acid (85:15:1, *v*/*v*/*v*) solvent system. Methanolic MnCl_2_ solution (0.63 g MnCl_2_·4H_2_O, 60 ml water, 60 ml methanol, and 4 ml concentrated sulfuric acid) was sprayed over the plate and kept for charring at 120 *°*C for 10 min. For the detection of FAMEs by TLC, the samples were spotted on TLC plate and developed by the dual-solvent system, firstly with hexane/*tert*-butyl methyl ether/acetic acid (50:50:0.5, *v*/*v*/*v*), and then air dried, redeveloped to 8 cm from the origin with hexane/*tert*-butyl methyl ether/acetic acid (97:3:0.5, *v*/*v*/*v*). The spots were visualized by spraying sulfuric acid 50% (*w*/*w*) followed by charring the plates at 135 °C. The extracted lipids from the water mold were further analyzed by FTIR spectrometer (Thermo Nicolet NEXUS, Maryland, USA).

### Gas chromatography-mass spectrometry analysis of fatty acid methyl esters

The products of transesterification were analyzed by GC-MS (Agilent, Santa Clara, CA, USA). A capillary column (DB- 5MS) with dimensions 30 m × 0.25 mm ID and 0*.*25 μm film thickness was used. The conditions of GC-MS were as follows: the samples were injected in split-less injection mode (1 μl at 250 °C), using helium as a carrier gas at a rate of 1 ml/min. The column temperatures were kept according to the following temperature program: from 50 °C (held for 1.5 min) to 180 °C (25 °C/min) followed by a further increase to 220 °C for 1 min (10 °C/min) and finally from 220 to 250 °C (15 °C/min, held for 3 min). The mass spectra of FAMEs were recorded with electron ionization (70 eV) in scan mode (50–600 m/z).

### Statistical analysis

All batch cultivation experiments were performed in triplicates, whereas the lipid estimation and GC-MS analysis were done in duplicates. The data were expressed as mean ± standard deviation and were analyzed with one-way analysis of variance (ANOVA) using Microsoft Office Excel 2016, with *p* values of < 0.05 being regarded as significant.

## Results and discussion

### Identification and characterization of isolated water mold

**Fig. 2:**
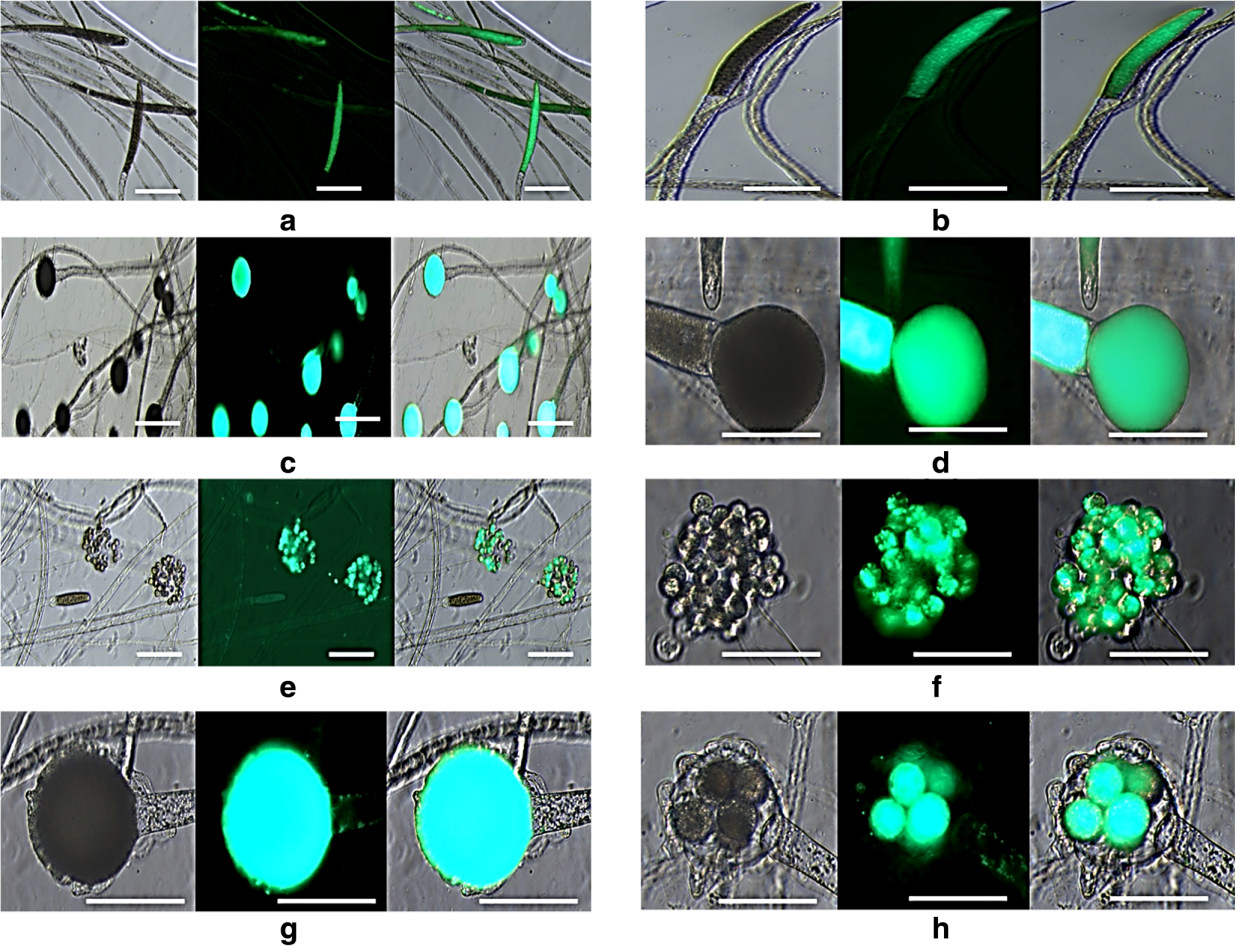
Representative fluorescence images of *A. diffusa* living cells stained with BODIPY showing TAG accumulation in cellular compartments. **a**, **b** Mature zoosporangium. **c**, **d** Mature oögonium. **e**, **f** Zoosporangial structure in *A. diffusa* empty zoosporangia in cymose manner. **g** Oögonium with monoclinous and diclinous antheridial branches. **h** Spherical oögonia with different types of antheridial attachments in *A. diffusa.* Scale bar represents 20 μm

The specific characteristics (the formation of sporangia; development, orientation, specific shape, and size of oögonia/antheridia; the structure of oöspores; and in the case of *Achlya,* the mode of formation and discharge of sporangia and the extent of the swarmer cycle) have been studied to identify the species. Morphological identification of the isolated mold was done according to Coker and Khulbe (Coker 1923; Khulbe 1980). All the morphological characterization of *A. diffusa* was performed with the help of fluorescence microscopy bright field images and are presented in Fig. 2 (left panel). The vegetative thallus or hyphae of the isolated culture was slender, sparingly branched, thin, and multinucleate. However, the differences in hyphae were clearly visible where reproductive structures (sporangia, antheridial cells, and oögonia) were damaged at the top of hyphae. Gemmae were produced in abundance, filiform, branched, single or catenulate, terminal or intercalary and of variable shapes and sizes. They were usually disrupted and germinated to form new hyphae. Asexual reproduction in *Achlya* was assisted by biflagellate zoospores (Plano spores) formed in clavate, or by filiform zoosporangia, which was abundant and was 200–820 μm long and 25–30 μm in diameter (Fig. 2a, b). Zoosporangia usually appear prior to the development of the sexual reproductive organs, and it has been reported that both sexual and asexual reproductive structures are on the same plant. As in the Saprolegnia genus, vegetative and asexual reproductive structures are different from each other, and these structures are considered as diagnostic or delimiting features (Fig. 2c, d). “Male” and “female” reproductive elements offer morphological characteristics to differentiate the various species of the Saprolegnia genus. Furthermore, male antheridium was formed, divided into two parts, the antheridial branch (slender hyphal portion) and the antheridial cell (terminal portion). The antheridial cell was separated to the branch by a septum. The antheridia were formed at the same time of oögonia appearance, and they made contact with the oögonial wall (Fig. 2g, h). The female gametangium or the oögonium was usually lateral in position to hyphal branch and usually 30–120 μm spherical or pyriform in shape. The oögonium was cut off from its concomitant stalk or hypha by a transverse septum. Two to eight oöspheres (eggs) were developed after the process of oogenesis, and they measured 15–25 μm in diameter. The sexual reproduction in oomycete is usually accomplished when the antheridial cell enters into the oögonium through a fertilization tube or cytoplasmic contact and fertilizes an oösphere. However, the identification and classification of *Saprolegnia* are problematic even after molecular characterization by different techniques due to the morphology of asexual zoosporangia and the way to produce spores, which determine the delineation of genera in the Saprolegniales (Vega-Ramírez et al. 2013). In the present study, BODIPY_505/515nm_ was used as a lipophilic bright greenish fluorescent dye that required a minimum staining time of 5 min with low quantity of dye. It was used to locate neutral lipids in a cellular compartment of *A. diffusa* via live cell imaging protocol (Fig. 2 right panel). Several methods such as GC-FID, GC-MS, TLC-FID, MALDI-TOF-MS, NMR, and HPLC have been introduced to identify and quantify the lipid classes of microbial origin. Chromatographic techniques to identify and quantify the microbial lipid classes require many complicated processes, including solvent extraction of lipids (Perona and Gutierrez 2004). To overcome this problem, fluorescence microscopy for TAG estimation has recently been introduced, which has many advantages over chromatographic methods. There are several types of dyes used for the same purposes; however, BODIPY_505/515_ nm is the lipophilic stain that can be used to quantify the neutral lipid accumulation within the cells by fluorescence microscopy (Patel et al. 2014). Sudan black B is also used as a staining dye to detect the lipid in the microorganisms, but it cannot distinguish between the lipid classes.

### Biomass and lipid production using sugarcane bagasse semi-solid fermentation

**Fig. 3:**
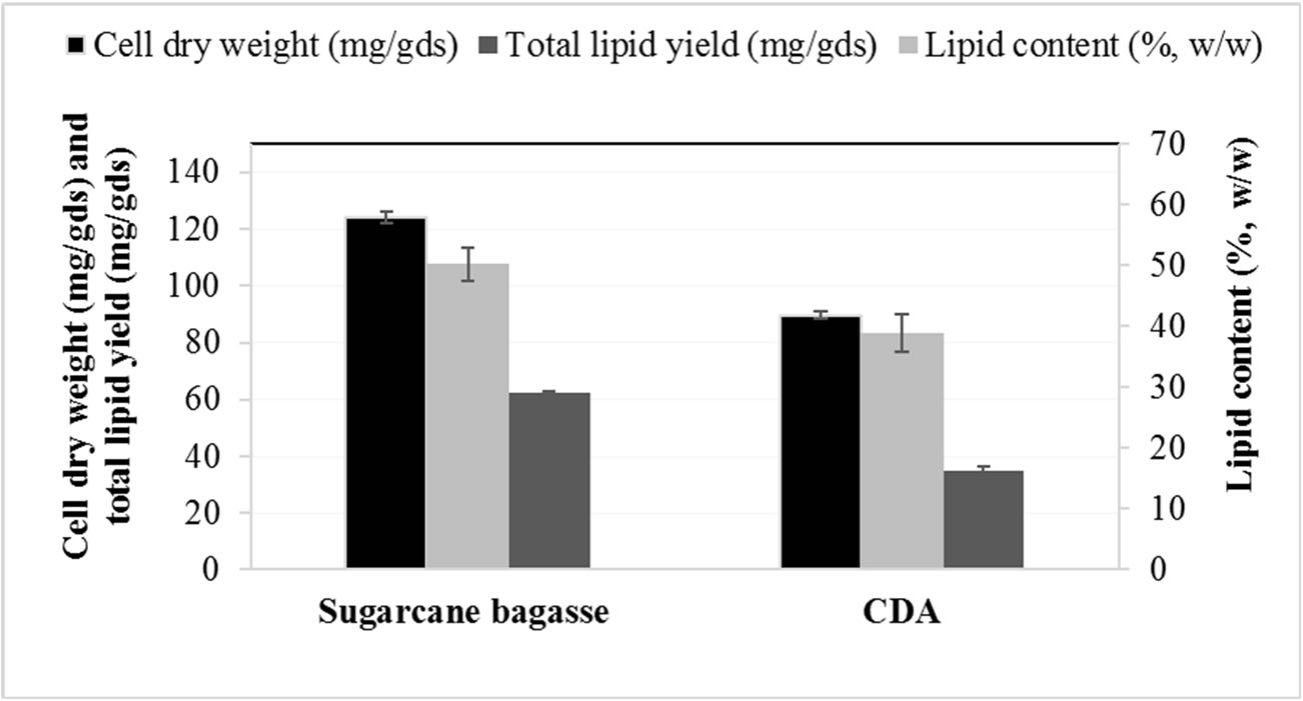
Graph showing cell dry weight (CDW) in mg/gds, total lipid yield (mg/gds), and lipid content (%) of *A. diffusa* grown on sugarcane bagasse and Czapek-Dox agar media, which was used as a control for lipid production

**Fig. 4:**
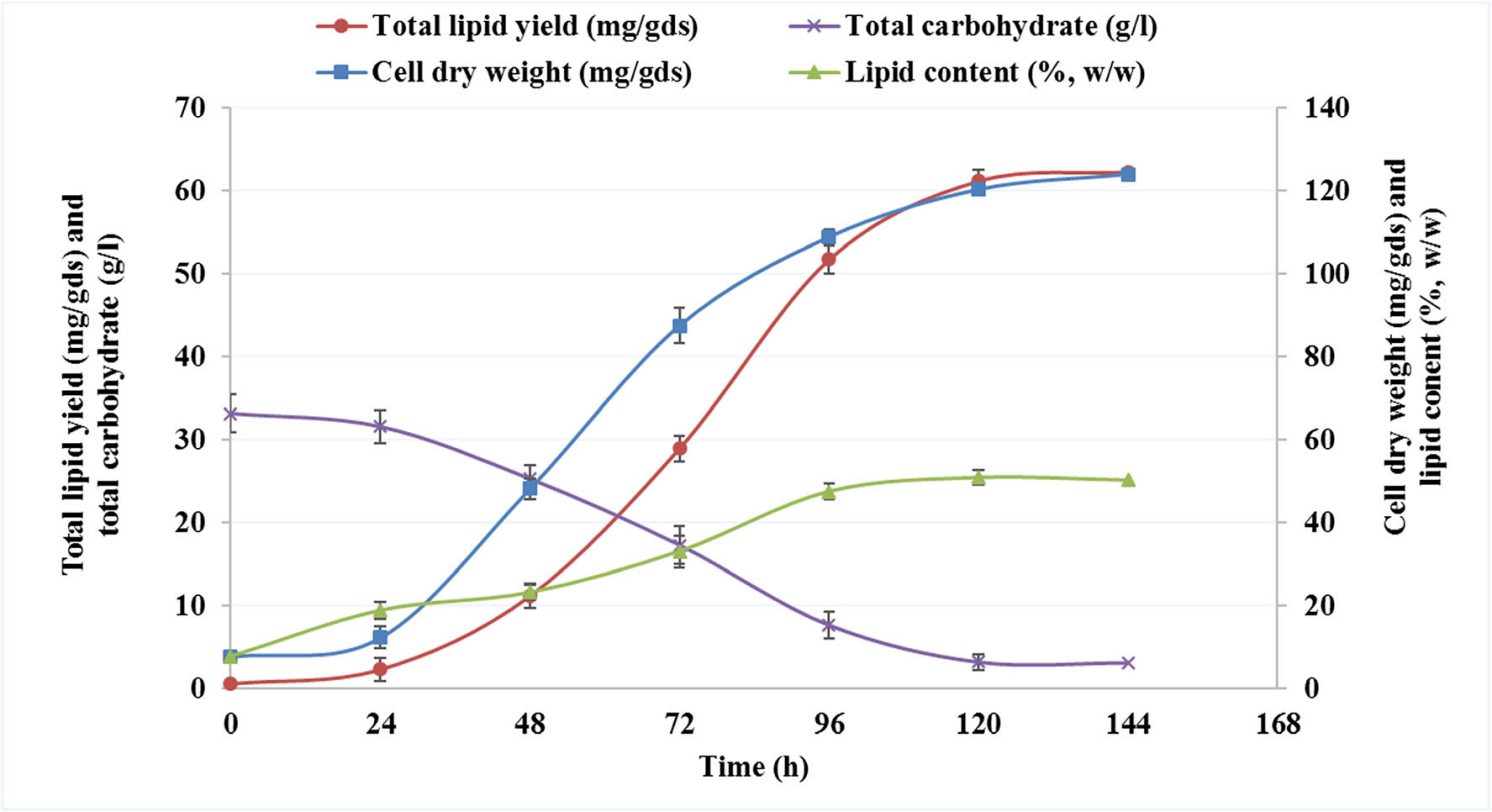
Time course experiment of cell dry weight, total lipid yield, and lipid content of *A. diffusa* grown on sugarcane bagasse for 144 h. The sampling was done in a regular time interval of 24 h

Solid-state fermentation is usually carried for microbial (fungal) growth, in which the solid substrates are provided to microorganisms without addition of water (Cheirsilp and Kitcha 2015). Several oleaginous molds such as *Mortierella isabellina* (Zhang and Hu 2012)*, Microsphaeropsis* sp. (Peng and Chen 2008)*, Aspergillus tubingensis* (Kitcha and Cheirsilp 2014)*, A. oryzae* (Hui et al. 2010), *Colletotrichum* sp., and *Alternaria* sp. (Dey et al. 2011) have been studied for their lipid accumulation ability and growth on solid substrates as reported in Table 1. In this study, a semi-solid fermentation (SSF) process is introduced by adding low amount of water to ensure the high fungal growth rate and easy substrate availability. The results of all batch cultivations of *A. diffusa* for cell dry weight (CDW) in mg/g dry substrate (gds), total lipid yield (mg/gds), and lipid content (%, *w*/*w*) are presented in Fig. 3. When sugarcane bagasse was provided to *A. diffusa* for growth, the CDW, total lipid yield, and lipid content were 124.03 ± 1.93 mg/gds, 62.34 ± 0.65 mg/gds, and 50.26 ± 1.76%, respectively, which is satisfactory considering that no sugarcane bagasse pretreatment took place. The commercialization of biodiesel production from oleaginous microorganisms is limited by the cost of feedstocks, as this accounts for 60–85% of the total production cost (Li et al. 2008). Researchers are interested in using agricultural residues as solid substrates, such as wheat and rice straw as well as its brane, soybean hull (Table 1), and easily hydrolysable carbon sources, such as sweet sorghum. However, to the best of our knowledge, sugarcane bagasse as feedstock in solid-state fermentation has not yet appeared in literature. The cell dry biomass and lipid content of this water were decreased to 89.54 ± 1.21 mg/gds and 38.82%, respectively, when cultivated on standard medium Czapek-Dox agar. The time course experiments of total carbohydrate utilization (g/l), CDW (mg/gds), and lipid yield (mg/gds) of *A. diffusa* grown on sugarcane bagasse for 144 h are presented in Fig. 4. Initially, after 24 h of growth, when sugarcane bagasse was provided as a solid medium, the CDW, total lipid yield, and lipid content were reported as 12.37 ± 1.09 mg/gds, 2.34 ± 0.12 mg/gds, and 18.92%, respectively, while only 4.78% carbohydrate was utilized by *A. diffusa*. After 48 h of growth, the cell dry weight and lipid content was increased exponentially to 120 h of cultivation period while the maximum utilization of carbohydrate (9.65 g/l) was reported between 72 and 96 h of growth (Fig. 4). However, the culture attained its stationary phase at 144 h due to 90.36 ± 2.3% consumption of total carbohydrate. It is necessary to harvest the cells for lipid extraction in early stationary phase of culture, because in late stationary phase, the stored lipid droplets would be cleaved by lipases, present in the cellular compartment, to compensate for the survival under nutrient-deficient condition (Ratledge 2004). Earlier, Economou et al. (2010) reported that the oleaginous fungus *Mortierella isabellina* produced more lipids (11 g/100 g dry weight of substrate) when grown under semi-solid conditions on sweet sorghum compared to the solid-state cultivation, demonstrating the superiority of the semi-solid-state cultivation over solid state in terms of lipid yield and quality (Economou et al. 2010). Moreover, oleaginous fungi *Aspergillus tubingensis* produced 31.1 ± 1.7 and 37.5 ± 2.2 mg/gds of lipid under solid-state cultivation on palm-pressed fiber and palm empty fruit bunches, respectively (Kitcha and Cheirsilp 2014). Another fungus, *A. oryzae* A-4 produced 36.6 mg/gds of lipid under optimized conditions and increased the lipid production to as high as 62.87 mg/gds on the 6th day of solid-state fermentation on wheat straw and bran mixture (Hui et al. 2010). Finally, two endophytic fungal isolates, *Colletotrichum* sp. (isolate DM06) and *Alternaria* sp. (isolate DM09) synthesized 68.2 and 60.32 mg/gds of lipid when grown on rice straw and wheat bran under solid-state fermentation (Dey et al. 2011).

**Table 1:** Comparative studies on lipid production of oleaginous molds growing on lignocellulosic biomass in solid-state fermentation condition

Oleaginous molds	Non-edible lignocellulosic biomass	Mode of Cultivation	Lipid production	References
*Aspergillus tubingensis*	Palm pressed fiber (PPF)	Solid-state fermentation	31.1 ± 1.7 mg/g dry substrate (gds)	Kitcha and Cheirsilp (2014)
	Palm empty fruit bunches (EFB)	Solid-state fermentation	37.5 ± 2.2 mg/gds	
*Microsphaeropsis* sp.	Steam-exploded wheat straw (SEWS) and wheat bran (WB)	Solid-state fermentation	42 mg/gds	Peng and Chen (2008)
*Colletotrichum* sp. (isolate DM06)	Rice straw and wheat bran	Solid-state fermentation	68.2 mg/gds	Dey et al. (2011)
*Alternaria* sp. (isolate DM09)	Rice straw and wheat bran	Solid-state fermentation	60.32 mg/gds	
*A. oryzae* A-4	Wheat straw and bran	Solid-state fermentation	36.6 mg/gds	Hui et al. (2010)
*Mortierella isabellina*	Soybean hull	Solid-state fermentation	47.9 mg/gds	Zhang and Hu (2012)
*A. diffusa*	Sugarcane bagasse	Semi-solid-state fermentation	62.34 ± 0.65 mg/gds	This study
	Czapek-Dox agar	Solid-state fermentation	34.76 ± 1.21 mg/gds

### Thin layer chromatography and Fourier-transform infrared of extracted lipid from sugarcane bagasse grown *A. diffusa*

**Fig. 5:**
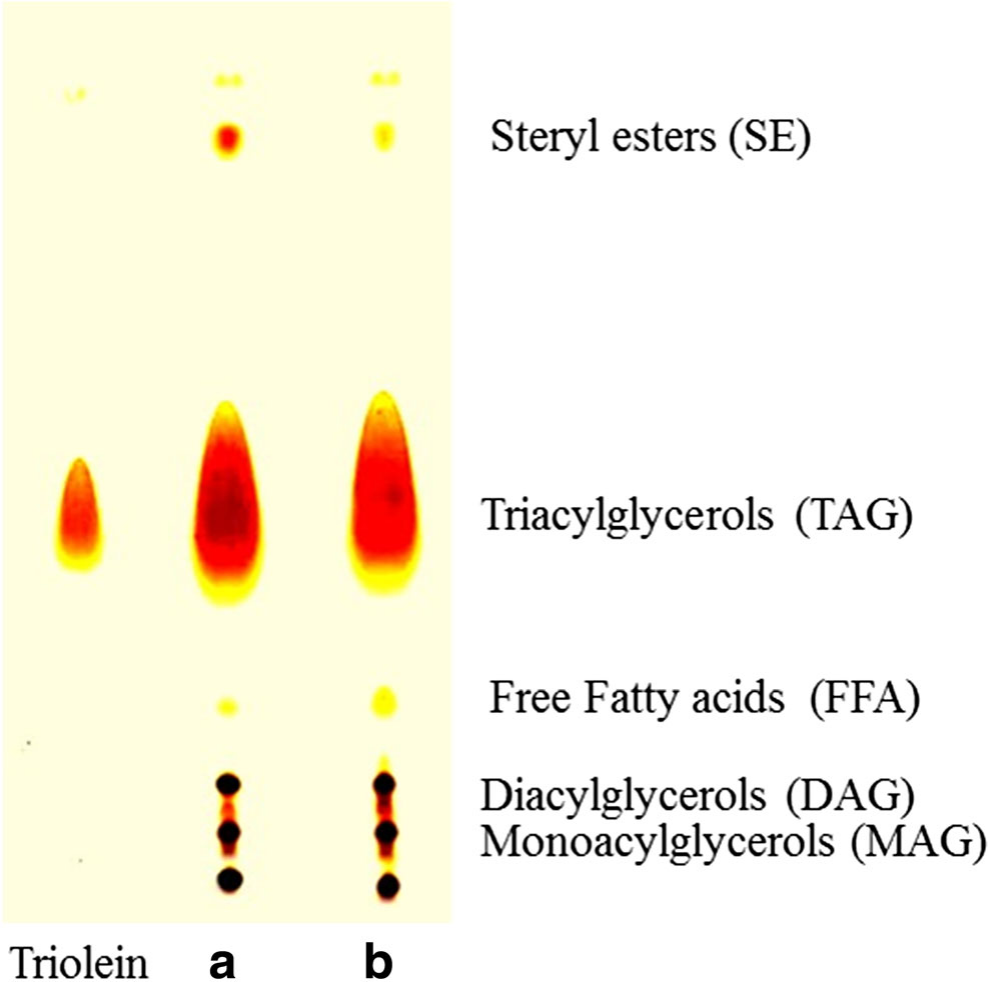
TLC chromatogram of extracted lipid from *A. diffusa* grown on **a** sugarcane bagasse and **b** Czapek-Dox agar media. Triolein is used as standard for the TAG (lane 1)

**Fig. 6:**
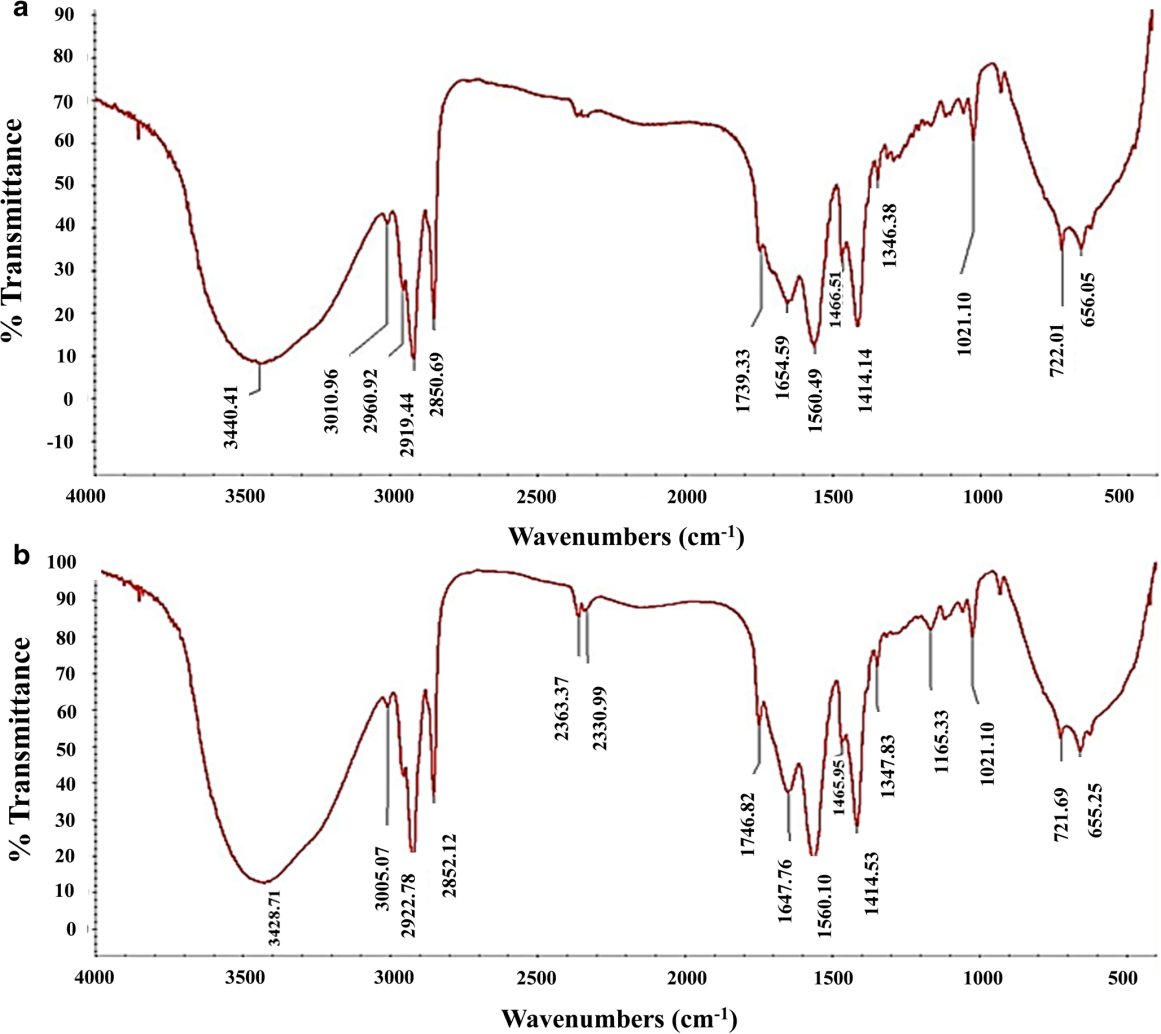
FTIR spectra of lipids extracted from *A. diffusa* grown on sugarcane bagasse (**a**) and in comparison with triolein (**b**)

After lipid extraction from the water mold, prior to the analysis of their contents, TAG separation is necessary. The preferred methods for separation of TAG are solid-phase extraction, preparative HPLC and TLC (Patel et al. 2016). The most known, simple, rapid, and single-step method for separation of TAGs is TLC, in which all contents of extracted lipids are easily resolved (Fedosov et al. 2011). Diethyl ether, present in the mobile phase, is responsible for the resolution of the TAG content, while the free fatty acids (FFAs) migrate due to hexane and acetic acid. The chromatogram for total lipid separation is presented in Fig. 5 where triolein was used as a control for TAG. Total lipids were resolved as TAG, diacylglycerols (DAGs), monoacylglycerols (MAGs) and steryl esters (SEs). High amount of TAG, present in both samples, shows its efficiency regarding the transesterification reaction as 1 mol TAG generates 3 mol of biodiesel and 1 mol of glycerol in a complete reaction. Fourier-transform infrared (FTIR) spectroscopy was also used to identify the TAG groups present in total lipid after extraction. The FTIR spectra of lipid extracted from sugarcane bagasse grown *A. diffusa* showed transmittance spectral similarities with triolein used as standard (Fig. 6). The specific molecular group present in total extracted lipid absorbed a specific range of infrared waves, and obtained bands were used for identification (Murdock and Wetzel 2009). The spectral region between 3950 and 3450 cm^−1^ did not show any band, which is an indication of absent hydroxyl and amine groups. All TAG groups were presented in the second spectral region between 3100 and 2850 cm^−1^ showing similar bands to triolein (Ami et al. 2014).

### Fatty acid profiles of water mold grown in sugarcane bagasse and Czapek-Dox agar and estimation of biodiesel properties on the basis of fatty acid profile

Conversion of oil from oleaginous microorganisms into biodiesel mainly involves four crucial steps: cultivation, harvesting cells, lipid extraction, and transesterification. One important bottleneck of biodiesel production from oleaginous microorganism is the lipid extraction expenses (Liu et al. 2012). Oleaginous microorganisms usually synthesize a vast majority of lipid classes, which are not suitable for biodiesel production; the fatty acid methyl esters (FAMEs) are produced by only those lipids that have fatty acid ester linkages and free fatty acids (Ratledge 2004; Papanikolaou and Aggelis 2011). *A. diffusa* grown on sugarcane bagasse showed higher lipid accumulation than on Czapek-Dox agar CDA (Fig. 3). For the fatty acid profile of the sugarcane bagasse grown *A. diffusa,* in situ transesterification (IST) and indirect transesterification (IDT) approaches were evaluated. The analysis of FAME was performed by GC-MS and the results are presented in Table 2. When IST method was used for transesterification, the fatty acid methyl esters consist of mainly 2.54 ± 0.12%, myristic acid (C_14:0_); 13.45 ± 0.32%, palmitic acid (C_16:0_); 5.02 ± 0.22%, stearic acid (C_18:0_); 26.56 ± 0.76%, arachidic acid (C_20: 0_); 45.54 ± 0.65%, oleic acid (C_18: 1_); and 4.09 ± 0.98%, linoleic acid (C_18:2_) along with 2.09 ± 0.26%, α-linolenic acid (C_18:3_). IST method showed higher amount of monounsaturated fatty acids (45.54%) as well as total fatty acid content than that was obtained after IDT method (39.46%). Due to the presence of high saturated fatty acids along with monounsaturated fatty acids, IST method proved efficiency toward transesterification method. IST method also showed high amount of long-chain arachidic acid methyl ester (C_20:0_), which indicates the potential fuel properties in terms of high cetane number. On the contrary, the fatty acid obtained from CDA grown cells showed 2.74 ± 0.51%, lauric acid (C14:0); 1.73 ± 0.42%, myristic acid (C_14:0_); 22.87 ± 0.34%, palmitic acid (C_16:0_); 23.32 ± 0.72%, stearic acid (C_18:0_); 2.12 ± 0.12%, arachidic acid (C_20: 0_); 31.63 ± 0.31%, oleic acid (C_18: 1_); and 0.79 ± 0.11%, linoleic acid (C_18:2_) along with 1.23 ± 0.41%, α-linolenic acid (C_18:3_).

**Table 2:** The total percentage of fatty acid methyl esters (FAMEs) produced by *A. diffusa* grown on sugarcane bagasse as well as Czapek-Dox agar media. The lipids obtained from sugarcane bagasse grown culture were transesterified with two different methods such as indirect transesterification (two-step transesterification, IDT) and in situ transesterification (IST)

FAMEs composition (%)		*A. diffusa* grown on sugarcane bagasse				*A. diffusa* grown on CDA	
		IST		IDT		IST	
SFA	Lauric acid methyl ester (C_12:0_)	ND	47.57%	1.10 ± 0.15	49.3%	2.76 ± 0.51	52.8%
	Myristic acid methyl ester (C_14:0_)	2.54 ± 0.12		2.71 ± 0.34		1.73 ± 0.42	
	Palmitic acid methyl ester (C_16:0_)	13.45 ± 0.32		16.03 ± 0.21		22.87 ± 0.63	
	Margaric acid methyl ester (C_17:0_)	ND		ND		ND	
	Stearic acid methyl ester (C_18:0_)	5.02 ± 0.22		8.73 ± 0.29		23.32 ± 0.72	
	Arachidic acid methyl ester (C_20:0_)	26.56 ± 0.76		20.73 ± 0.12		2.12 ± 0.12	
MUFA	Oleic acid methyl ester (C_18:1_)	45.54 ± 0.65	45.54%	39.46 ± 0.45	39.46%	31.63 ± 0.31	31.63%
PUFA	Linoleic acid methyl ester (C_18:2_)	4.09 ± 0.98	6.18%	1.30 ± 0.42	3.01%	0.79 ± 0.11	2.02%
	α-Linolenic acid methyl ester (C_18:3_)	2.09 ± 0.26		1.71 ± 0.17		1.23 ± 0.41	

*SFA* saturated fatty acids, *MUFA* monounsaturated fatty acids, *PUFA* polyunsaturated fatty acids, *ND* not detected

The biodiesel production at large scale still faces problems in regard to high costs involved in feedstocks and fatty acid methyl esters conversion routes. In the conventional route, biodiesel is usually produced by transesterification reaction in which oils extracted from feedstocks react with methanol in the presence of suitable catalysts (Patel et al. 2018a). The lipids synthesize inside the cellular compartment of oleaginous microorganisms; hence, extraction of lipids involves various steps, including disruption of cells, which finally increase the overall production cost (Patel et al. 2018b). In the present study, we tried to solve this problem by in situ transesterification reaction in which prior lipid extraction from harvested biomass is not required. This was only a preliminary experiment to check the difference in fatty acid profile obtained from IST and IDT methods. However, in situ transesterification reaction depends on several variables such as alcohol to biomass ratio, reaction time, and temperature and moisture contents of feedstocks, which should be further optimized with this water mold. The fuel properties were theoretically estimated by using fatty acid profiles of water mold grown on various substrates, and data are presented in Table 3. The properties of biodiesel as a fuel are greatly influenced by the quality of fatty acids present in original oils (Knothe and Razon 2017). It has been reported that the cetane number, heat of combustion, melting point, and viscosity of biodiesel are improved with increasing the chain length of fatty acids and is declined with increasing unsaturation in fatty acids (Knothe 2010; Patel et al. 2017a). Moreover, biodiesel fuels, derived from fats or oils with significant amounts of saturated fatty compounds, will display higher cetane number and oxidative stability while the unsaturation in the fatty acids supports its cold flow plugging properties. Density and kinematic viscosity are important parameters to determine the flow and energy of any fuel. It has been reported that denser biodiesel has more energy than petroleum diesel, and the kinematic viscosity of petroleum diesel is 10 to 15% lower than that of biodiesel due to its smaller molecular weight and structure. The long saturation factor (LCSF) of the biodiesel obtained from water mold grown in sugarcane bagasse (after IST and IDT methods) were 30.42 and 26.70, respectively, and the corresponding kinematic viscosities were 4.34 and 3.84 mm^2^/s, respectively (Table 3). While, the kinematic viscosity of biodiesel obtained from water mold grown in CDA was 3.40 mm^2^/s, which does not follow the lower limit setup by EN-14214. The density of biodiesel obtained from water mold grown in sugarcane bagasse (0.87 g/cm^3^ after IST method) fulfills the criteria set up by international standard, whereas the biodiesel obtained after IDT method applied for water mold grown in sugarcane bagasse and CDA were 0.80 and 0.75 g/cm^3^, respectively. These values were lower than the limit setup by EN-14214 (Table 3). The low temperature performance of biodiesel is considered an important factor, and it is usually calculated by one of the three tests such as cloud point, pour point, and cold filter plugging point (Hoekman et al. 2012). The pour point of any fuel is the lowest temperature at which it can easily flow, and below this temperature the liquid loses its flow characteristics. Data shows that biodiesel obtained from water mold grown in sugarcane bagasse (after IST and IDT methods) has relatively good pour point (− 4.56 and − 3.84 °C) compared to that obtained from CDA grown culture (0.82 °C). Moreover, other properties of all biodiesel samples listed in Table 3 follow the criteria determined by both international standards ASTM-D6751 and EN-14214. Therefore, it is necessary to control the fuel properties by optimizing the ratio of both saturated and unsaturated fatty acids and the efficiency of any fuel cannot be described by considering a single parameter (Patel et al. 2017a).

**Table 3:** Theoretical estimation of biodiesel properties based on the fatty acid profiles of *Achyla diffusa* grown on sugarcane bagasse as well as Czapek-Dox agar media and in comparison, with international standards

Biodiesel properties	Units	*A. diffusa* grown on sugarcane bagasse		*A. diffusa* grown on CDA	Biodiesel Standards	
		IST	IDT	IST	ASTM D6751	EN 14214
					Limits	Limits
Long-chain saturation factor	–	30.42	26.70	16.07	–	–
Oxidative stability, 110 °C	h	21.67	41.77	60.97	3 min	6 min
Density	g/cm^3^	0.87	0.80	0.75	–	0.86–0.90
Cold filter plugging point	°C	79.08	67.40	34.00	–	–
Cloud point	°C	2.08	3.4	7.0	–	–
Pour point	°C	−4.56	−3.09	0.82	–	–
Cetane number	–	61.84	66.35	69.40	47 min	51 min
Kinematic Viscosity	mm^2^/s	4.34	3.84	3.40	1.9–6.0	3.5–5
Saponification value	mg KOH/g-oil	197.00	184.32	178.47	0.50 min	0.50 min
Iodine value	mgI_2_/100 g	54.08	42.52	33.24	–	120 max
High heating value	MJ/kg	39.42	36.38	34.13	–	–

*–* not reported, *Min* minimum, *Max* maximum, *IST* in situ transesterification, *IDT* indirect transesterification

## Conclusions

The present study shows for the first time the use of a novel water mold, *A. diffusa*, for microbial oil production. *A. diffusa* grown on waste sugarcane bagasse as a semi-solid medium accumulated 62.32 ± 0.65 mg/gds total lipid with 50.26% lipid content. The fatty acid profile of lipids from *A. diffusa* suggests that it could be explored as a potential oleaginous microorganism for biodiesel production. Moreover, it can be integrated with water remediation processes as it has great capability to decay the organic matter present in water bodies. Several other factors affecting the growth and lipid accumulation of this water mold, such as C/N ratio, moisture content in semi-solid-state fermentation, inoculum size, and addition of nutrients including the lignocellulolytic activity of this mold should be further studied as it can grow on lignocellulosic waste directly without the need of enzyme addition (e.g., cellulases).
